# Dataset for weld seam analysis and discontinuity prediction in laser beam welding scenarios

**DOI:** 10.1016/j.dib.2025.111381

**Published:** 2025-02-10

**Authors:** Dominik Walther, Leander Schmidt, Klaus Schricker, Christina Junger, Jean Pierre Bergmann, Gunther Notni, Patrick Mäder

**Affiliations:** a Technische Universität Ilmenau, Data-intensive Systems and Visualization Group (dAI.SY), Max-Planck-Ring 14, 98693 Ilmenau, Germany; b Technische Universität Ilmenau, Production Technology Group, Gustav-Kirchhoff-Platz 2, 98693 Ilmenau, Germany; c Technische Universität Ilmenau, Quality Assurance and Industrial Image Processing Group, Gustav-Kirchhoff-Platz 2, 98693 Ilmenau, Germany; d Fraunhofer Institute for Applied Optics and Precision Engineering IOF Jena, Einstein-Str. 7, 07745 Jena, Germany; e Friedrich Schiller University, Faculty of Biological Sciences, Fürstengraben 1, 07743 Jena, Germany; f BTU Cottbus-Senftenberg, Chair of Joining and Welding Technology, Konrad-Wachsmann-Allee 13, 03046 Cottbus, Germany

**Keywords:** Laser Beam Butt Welding, Inductive Probes, LWIR Camera, Time Series, Images, Thin Steel Sheets, Classification, Regression

## Abstract

Laser beam welding can produce narrow, high-quality welds in various industrial joining processes. The thermal expansion and contraction of the metal during the weld results in the displacement of the sheets. That leads to the formation of joint gaps and subsequent to a process interruption. This behavior has only been analyzed to a limited extent and causes manufacturers to rely on heavy clamping systems rather than using more flexible fixtureless approaches. Due to the time-consuming and costly nature of recording and producing erroneous weld seams, such recordings and datasets are rarely available in this area. This often limits the research towards adaptable fixtureless welding setups. Because of this, we present a multi-modal dataset consisting of 100 recorded welds that tracks the metal sheets movement. The developed setup enables the determination of boundary conditions for fixtureless welding. Two types of sensors record the welding process. First, three inductive probes are applied to record the metal sheets` movement and second, a long-wave infrared (LWIR) camera records changes in the thermal radiation field. Two different welding speeds and laser powers were used to produce a variety of welds. The dataset can be used for data-driven algorithms to predict the metal movement, analyze the thermal radiation field, or develop quality control methodologies.

Specifications Table

**Table:** 

Subject	Manufacturing Engineering, Applied Machine Learning, Metals and Alloys
Specific subject area	Quality monitoring for laser beam butt welding. Prediction and classification of weld failures on multi-modal data*.*
Type of data	Time Series, Table, Image, Raw, Preprocessed, Labeled
Data collection	The sheets were placed initially in a zero-gap position at the start of each welding process. One sheet is clamped, while the second sheet is movable in the X-Y plane. A Trumpf TruDisk 5000.75 disk laser was used for each weld. The weld was recorded with two different sensor types. The time series data were measured with three inductive probes of type Millimar P2010 M. The images and video sequences were recorded with a long-wave infrared camera of type VarioCAM HD head 800.
Data source location	Institution: Technical University of Ilmenau City: Ilmenau Country: Germany
Data accessibility	The data is publicly available under the link provided below. Repository name: Figshare Direct URL to data: https://doi.org/10.6084/m9.figshare.27877413.v1
Related research article	D. Walther, L. Schmidt, K. Schricker, C. Junger, J. P. Bergmann, G. Notni, P. Mäder, Automatic detection and prediction of discontinuities in laser beam butt welding utilizing deep learning, Journal of Advanced Joining Processes 6 (2022) 100119. https://doi.org/10.1016/j.jajp.2022.100119

## 1 Value of the Data

• The data presented in this paper provide valuable insights into the formation and time- dependent behavior of joint gaps when welding thin steel sheets. Researchers can utilize the dataset to further understand and analyze the behavior of the metal movement.
• Researchers can utilize the annotated dataset to train and evaluate data-driven laser beam welding monitoring algorithms. The dataset provides multi-modal recordings from inductive probes to measure the metal sheets’ movement and long-wave infrared camera (LWIR) recordings that track the thermal radiation.
• Manufacturers could train machine learning methods on this dataset to predict and monitor welding processes. Researchers and scientists could gain more information on the welding process with this dataset and develop new approaches to predict and analyze welds.
• The dataset contains multi-modal process recordings of 100 welds, that can be utilized for 13 forecasting purposes, such as gap prediction and classification tasks like quality monitoring. Experiments with this dataset can give insights into the system behavior and provide a better understanding of the laser beam welding process for thin steel sheets.
• The dataset can be utilized for transfer learning [11] if weld parameters such as the material or laser configuration change. The findings from the proposed dataset can be transferred to other welding configurations if the gap characteristics are similar to the ones present in this dataset. This enables a faster and better training of newly developed methods. Researchers can achieve better results with less process-specific data. This reduces the experimental effort to acquire new process-specific data. A detailed discussion about the influence of different scenarios is presented in [8].
• The raw and processed data supports researchers in replicating experiments and ensures the transferability of their approaches. In specific, the dataset enables researchers to create baselines, evaluate newly developed techniques, test, and validate their methods.

## 2 Background

Laser beam welding is one of the state-of-the-art methods for advanced joining processes and is applied in many industrial tasks [1]. As the demands on product quality continue to increase, the quality of weld seams is of crucial importance to manufacturers. Achieving these standards often requires the utilization of advanced data-driven techniques for effective quality control and process monitoring. Especially deep learning is often the technique of choice for such tasks [6]. However, these techniques require sufficient data to learn and understand complex system behaviors. Recording data is usually an expensive and time-consuming task. Researchers and manufacturers are often limited by the amount of data available [2]. Welding processes are costly in terms of recording erroneous samples. But especially these samples are required for an efficient deep learning training. As a result, sufficient data to train advanced data-driven approaches is often lacking in this area. Therefore, we want to share our dataset with researchers and scientists, allowing them to train and evaluate new approaches that advance this field of research. This dataset may also enable them to extend existing datasets. This could improve data-driven methods developed and evaluated on their data.

## 3 Data Description

**Fig. 1:**
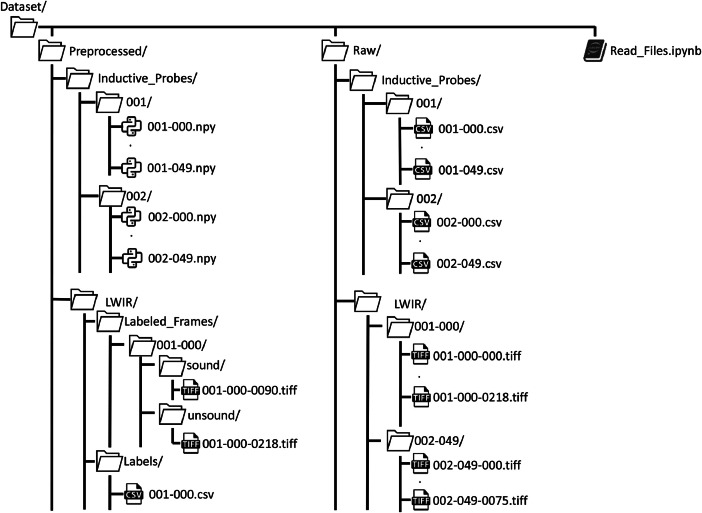
Folder structure of the proposed dataset. The dataset is separated into preprocessed and raw files and includes an IPython notebook with exemplary code to read and visualize the recordings.

**Fig. 2:**
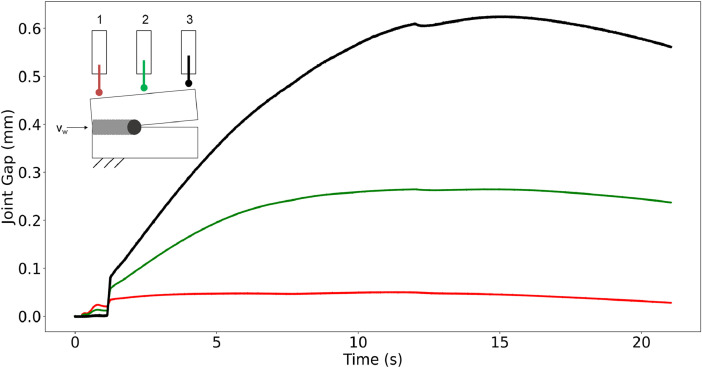
Exemplary plot of an inductive probe recording. The probes are placed at the beginning (red), the center (green), and the end (black) of the metal sheet. Figure adapted from [8].

The dataset is separated into raw and preprocessed as depicted in Fig. 1. **Raw:** The *Raw* folder contains all files recorded from the sensors without any modification to the data. In case of the LWIR camera, the images were cropped before being transmitted to focus on the region of interest (see Section Experimental Design, Materials and Methods). Some of the images may contain noise or artifacts. We included the raw data since we wanted to give researchers the opportunity to apply their preprocessing methods to optimize the data. As described in the Specifications Table, two sensor types were used to record each weld. The folders are separated for each sensor type. First, the folder of the inductive probes contains .csv files for each welding speed. Welds with a speed of 1 $\frac{m}{\text{min}}\;$are stored in folder *001* and for welding speed 5$\;\frac{m}{\text{min}}$ in folder *002*. Table 1 shows the general structure of each .csv file. The first columns of the *.csv* file contain the timestamp and the trigger signal of the weld. The trigger signal should be constant at 24 V and synchronize the start of the weld, the inductive probes, and the LWIR camera. The last three columns, named Probe 1–3, contain the inductive probes recordings at the beginning (red), center (green), and end (black) of the movable sheet as depicted in Fig. 2.

**Table 1:** Structure of inductive probe recordings.

Time (s)	Trigger (V)	Probe 1 (mm)	Probe 2 (mm)	Probe 3 (mm)
0	24	-0.00026811307	0.00039802567	-0.00029261451
0.001	24	-0.00026811307	0.00031954056	-0.00060655491
0.002	24	-0.00026811307	0.00039802567	-0.00099898048
.	.	.	.	.
.	.	.	.	.
.	.	.	.	.
21.043	24	0.028143495	0.23703063	0.56095439

The inductive probes track the total position change of the second moveable sheet in Y-direction (see Section 3). The gap widens as the sheet begins to move (see Fig. 2 at 0 s–15 s). This is due to local thermal expansion caused by the thermal expansion generated by the laser energy.

**Fig. 3:**
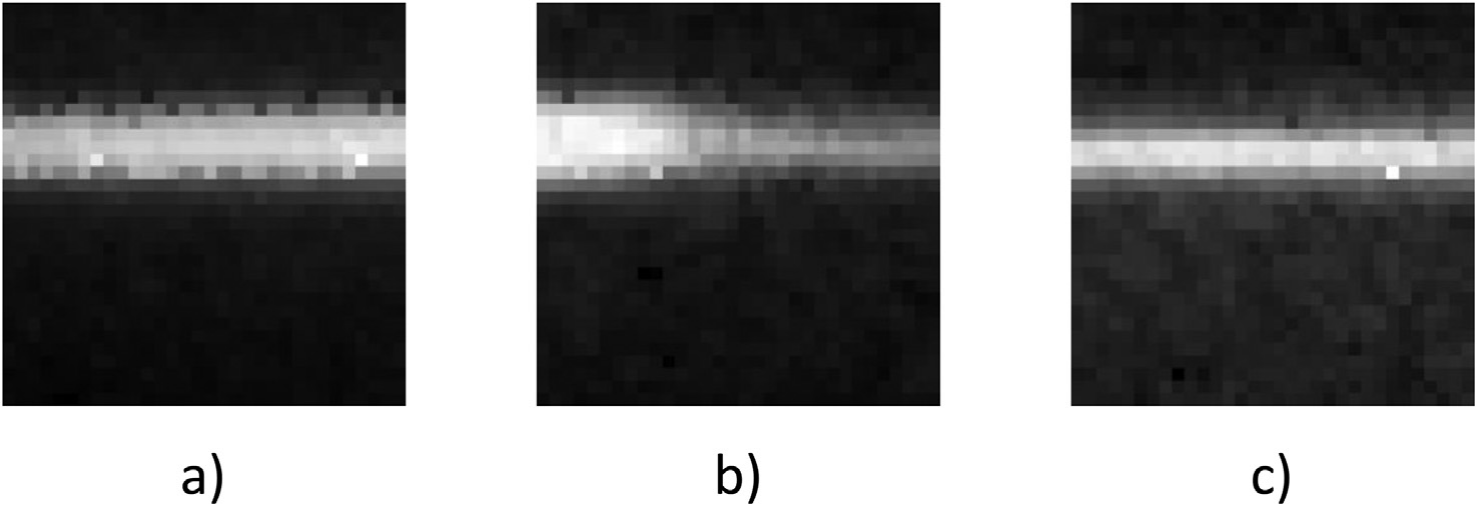
Exemplary images of the LWIR camera recordings. a) Thermal radiation field that indicates a sound fusion during the weld. b) Visible transition between sound and unsound fusion. c) Thermal radiation field that indicates an unsound fusion.

The gap narrows at the end of the weld when the weld cools down. The thermal contraction of the metal pulls the sheets together again and the gap starts to close (see Fig. 2 at 15 s–20 s). The length of the .csv files differs depending on the welding speed. The second folder contains the LWIR camera images. The images are stored for each weld individually in separate folders. The folders contain each recorded image as a .tiff file with a resolution of 32 × 32 px^2^. The images show the thermal radiation field. Fig. 3 visualizes some exemplary images. The camera recordings for the welds *001–015* and *002–047* are missing because the LWIR camera software froze at the beginning of the weld and did not record any images. We kept these folders in the dataset because the inductive probes did record the respective weld.

**Preprocessed:** The *Preprocessed* folder has the same structure as described before. The inductive probe recordings were filtered with a median filter to remove spikes that randomly occurred during the recording. The inductive probe recording was shortened at the beginning and the end to remove data that does not relate to the weld. This is because the trigger switched on to early or missed the end of the weld. The LWIR images are stored in folders with their respective label (Sound and Unsound) as shown in Fig. 1. Images in the beginning that do not show any thermal radiation are not included in these folders. But they are labeled in the *labels.csv* with a Beginning label, respectively. The labels are added as a .csv file where each LWIR camera image is annotated with a respective label. The label indicates whether the visible thermal radiation field shows a Sound or Unsound weld seam [8]. All files were labeled with Label Studio [7]. Images that contain artifacts or do not show any thermal radiation have been removed and are not included in the *Preprocessed* folder. This results in fewer images than included in the raw folder. This is especially the case at the weld's beginning and end due to the robot's movement and the placement of the camera behind the laser processing head [4].

## 4 Experimental Design, Materials and Methods

**Fig. 4:**
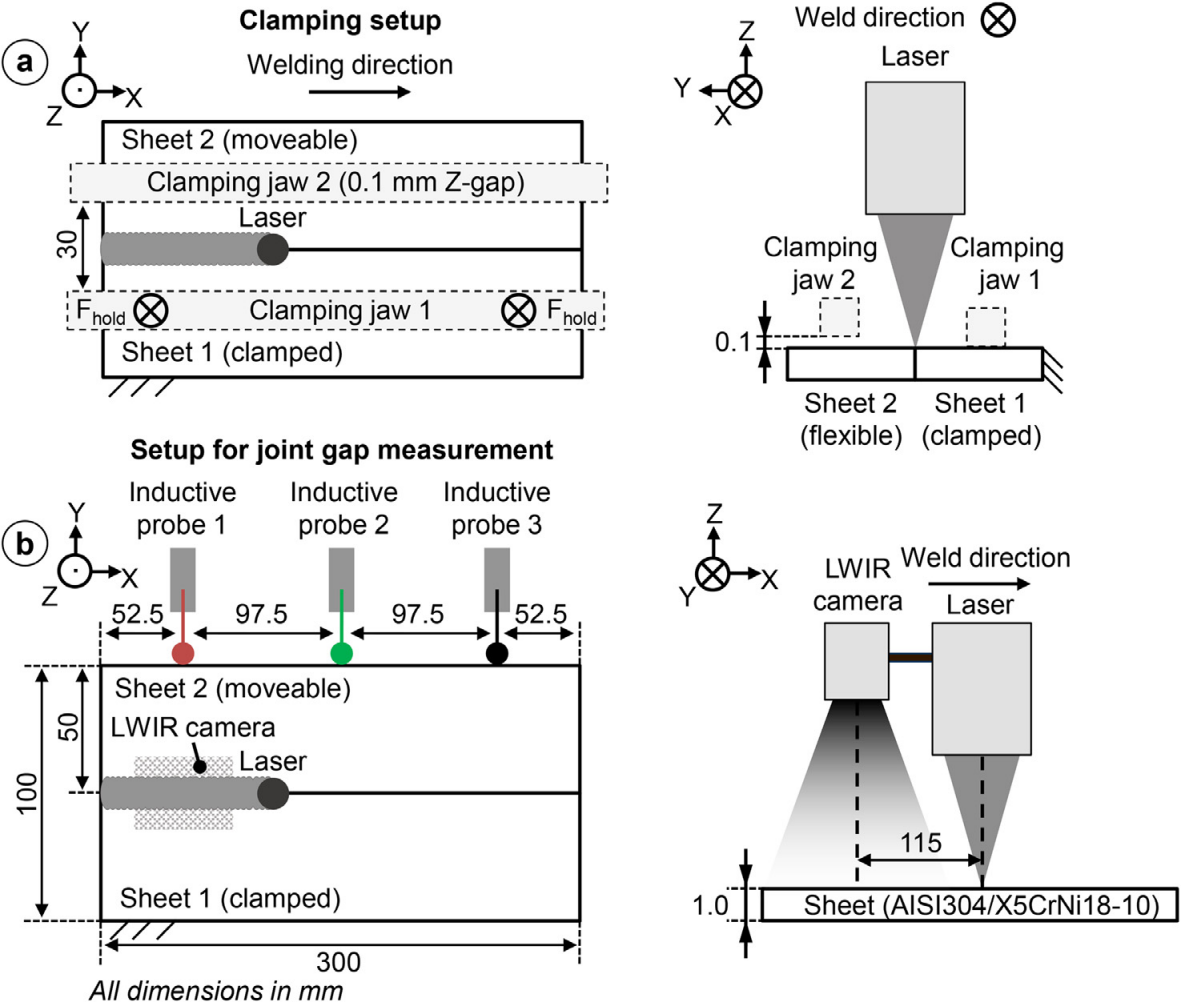
Schematic illustration of the experimental setup utilized to acquire the proposed data. a) illustrates the clamping system utilized to fix the first sheet and keep the second sheet movable. b) Shows the sensor placement of the inductive probes as well as the LWIR camera [8].

The experimental setup was previously described in several studies like [4,5,8,9] and is illustrated in Fig. 4. The dataset was recorded in two sessions with the described experimental setup. The setup was assembled before and disassembled after each recording session and the metal sheets were from two different batches. This allows us to capture possible variations in metal quality or environmental differences to portray a more realistic scenario. High-alloy steel sheets (material: AISI 304 / X5CrNi18-10 / 1.4301) were used to perform all welds. All sheets had a thickness of 1.0 mm and were cut with a laser. The metal sheets have a width of 50 mm and a length of 300 mm*.* All welds were performed in a butt joint with zero-gap configuration at the start of the welding process. The first sheet is held in place by a clamping jaw with a force of 1.2 kN. The second sheet could move freely in the X-Y-direction. The movement in Z-direction is limited to 0.1 mm by an additional clamping jaw. This setup is visualized in Fig. 4(a). We utilized two different welding speeds 1 $\frac{m}{\text{min}}\;$and 5 $\frac{m}{\text{min}}\;$with a laser power of 400 W and 1000 W, respectively. A Trumpf TruDisk 5000.75 disc laser is used. The laser has a focal diameter of 274 µm, a wavelength of 1030 µm, and a rayleigh length of 2.38 mm. The laser processing head is mounted to a six-axis robot (Kuka KR60-HA) to realize the laser movement. Two types of sensors, as depicted in Fig. 4(b), were utilized to record the joint gap formation during the weld. The movement of the second sheet in the Y-direction is measured by three inductive probes of type Millimar P2010 M. Probe 1 and Probe 3 were positioned 52.5 mm away from the cutting edges of the sheet. Probe 2 is positioned in the middle with a space of 97.5 mm of one another. The inductive probes offer a high measuring accuracy of 4 µm within a ± 2 mm measuring range and a 1000 Hz sampling frequency. We did not add any inductive probe to measure the movement in the X-direction since this movement does not have a major influence on the joint gap, which can cause the process to fail. The measurements of the inductive probes’ correlates to the movement of the movable second sheet and can be used to determine the joint gap formation. Fig. 2 shows an exemplary recording of the inductive probes. The distance between the laser and camera axis is determined at 115 mm, as shown in Fig. 4(b). The camera has a maximum resolution of 1,024 × 768 px^2^ and a sampling frequency of 30 Hz. We only used a window of 32 × 32 px^2^ to record the radiation field as close as possible to the laser spot and to focus on the weld seam. Other areas of the recording did not capture the thermal radiation and thus did not contain any valuable information about the weld seam. The camera can measure changes in the thermal radiation within a range between 250°C and 1700°C with an absolute accuracy of ± 1 K. We utilized the common EtherCAT (Ethernet for Control Automation Technology) [3] fieldbus for networking as it offers a high transmission speed and efficiency [10]. EtherCAT can be utilized for real-time computing, which is often necessary for automation technology. The first sheet (cf. Fig. 4) was placed and clamped to avoid movement. The second sheet was placed with zero gap next to the first sheet and the inductive probe heads were connected to the edge of the movable second sheet. Afterwards, the weld is initialized and the robot starts to move the laser processing head at the beginning of the sheets. As soon as the laser is automatically switched on at the start position the trigger signal switches to 24 V and the sensors start to record. The robot moves the laser processing head with the specified welding speed until the end of the sheet is reached. The trigger signal is set to zero when the laser turns off and the recording is finished. The sheets were removed from the welding setup and marked with a unique identifier for the welding speed as described in Section 2 and an ongoing number (cf. Fig. 1). The recordings were stored in folders with the same identifier on the respective sheet. Before the start of the next weld, the sensors were re-initialized and the next sheets were placed in the welding setup. This process was repeated until each weld was performed.

## Limitations

The dataset gives valuable insights and can be reused by manufacturers and researchers. However, it is important to discuss the limitations of the dataset. As described in Section 3, the LWIR camera is placed behind the laser. This causes the recording to show a delayed image of the radiation field. This can be critical in real-time applications, e.g., in a closed-loop control. Instead, the camera could be mounted in front of the laser to record an image of the laser spot without a delay. Initially, the camera was utilized to monitor the weld seam quality after the weld was finished [4]. We did not adjust the camera position to maintain consistency in the recording pipeline. The framerate of the LWIR camera was lower for the first recordings due to a human error during the initialization phase. Because of this, the first recordings contain fewer images than others. Nonetheless, do these images show the thermal radiation field of the laser with visible differences between sound and unsound fusion and can be used to train a classifier for quality monitoring.

## Ethics Statement

The current work does not involve human subjects, animal experiments, or any data collected from social media platforms. The authors have read and followed the ethical requirements for publication in Data in Brief.

## CRediT author statement

**Dominik Walther:** Writing – original draft, Visualization, Conceptualization, Data curation, Data annotation, Data preprocessing. **Leander Schmidt:** Writing – original draft, Visualization, Conceptualization, Data curation. **Klaus Schricker:** Writing – review, Conceptualization, Data curation. **Christina Junger:** Writing – review & editing, Conceptualization, Data curation. **Jean Pierre Bergmann:** Writing – review & editing, Funding acquisition. **Gunther Notni:** Writing – review & editing, Funding acquisition. **Patrick Mäder:** Writing – review & editing, Supervision, Funding acquisition.

## Data Availability

FigshareDataset for Weld Seam Analysis and Discontinuity Prediction in Laser Beam Welding Scenarios (Original data).
